# Association of Genetic, Environmental, and Nutritional Factors With Metabolic Phenotypes of Obesity: A Scoping Review

**DOI:** 10.1155/jobe/8472196

**Published:** 2025-07-02

**Authors:** Haniyeh Danesh Doost, Milad Nasiri Jounaghani, Roya Riahi, Motahar Heidari-Beni, Mohsen Hosseini, Fariborz Sharifianjazi, Roya Kelishadi

**Affiliations:** ^1^Faculty of Social Sciences (Unit of Health Sciences), Tampere University, Tampere, Finland; ^2^Department of Clinical Nutrition, School of Nutrition and Food Sciences, Isfahan University of Medical Sciences, Isfahan, Iran; ^3^School of Public Health, Epidemiology and Biostatistics Department, Isfahan University of Medical Sciences, Isfahan, Iran; ^4^Department of Nutrition, Child Growth and Development Research Center, Research Institute for Primordial Prevention of Non-Communicable Disease, Isfahan University of Medical Sciences, Isfahan, Iran; ^5^Department of Biostatistics and Epidemiology, School of Health, Isfahan University of Medical Sciences, Isfahan, Iran; ^6^School of Science and Technology, University of Georgia, Tbilisi, Georgia; ^7^Department of Pediatrics, Child Growth and Development Research Center, Research Institute for Primordial Prevention of Non-Communicable Disease, Isfahan University of Medical Sciences, Isfahan, Iran

**Keywords:** environment, genetic, nutrition, obesity, phenotype

## Abstract

**Background:** There are inconsistent findings regarding the different metabolic phenotypes of obesity and associated risk factors. This scoping review aims to provide a comprehensive summary of the literature that has evaluated the relationship between genetic, environmental, and nutritional factors and metabolic heterogeneity in obese and normal-weight individuals.

**Methods:** This scoping review was conducted according to the Preferred Reporting Items for Systematic Reviews and Meta-Analyses Extension for Scoping Reviews (PRISMA-ScR) guidelines. A literature search was conducted in Web of Science, the MEDLINE database (PubMed), Scopus, and Google Scholar up to the beginning of April 2024. All observational studies (cross-sectional, case-control, and cohort) were included.

**Results:** Ninety-two studies were included. Of these studies, 20, 38, and 20 were evaluated for the association between genetic, nutritional, and environmental factors with metabolic phenotypes, respectively. Genetic background could be a significant factor in obesity's metabolic phenotypes. Unhealthy dietary patterns, physical inactivity, improper sleep habits, smoking, and alcohol consumption could be related to an increased risk of metabolic phenotypes.

**Conclusion:** Environmental and nutritional factors can increase metabolic abnormalities. Metabolic phenotype categories are useful for predicting disease risk and for developing personalized diets and environmental interventions. These findings may help develop strategies to improve metabolic health.

## 1. Introduction

The global obesity epidemic has reached unprecedented levels, with over 1 billion people worldwide now living with obesity [1]. Obesity and overweight are correlated with a wide range of metabolic risk factors and chronic diseases, including insulin resistance, inflammation, dyslipidemia, hypertension, Type 2 diabetes mellitus (T2DM), cardiovascular disease (CVD), and metabolic syndrome (MetS) [2]. The MetS is a cluster of metabolic abnormalities that are associated with weight gain and obesity [3]. Also, metabolic disorders such as obesity and T2DM involve altered levels of adiponectin, which promotes insulin sensitivity and regulates glucose and fatty acid catabolism [4–6]. A recent study highlights the protective functions of adiponectin in obesity and diabetic complications, indicating its potential as a therapeutic target [7].

Obesity is strongly associated with the development of metabolic disorders, but there are distinct metabolic phenotypes among obese and normal-weight individuals that affect metabolic health [8–13]. Among obese individuals, two primary categories are often discussed: metabolic healthy obesity (MHO) and metabolic unhealthy obesity (MUHO) [8, 9]. MHO refers to individuals with obesity based on BMI criteria but who maintain relatively healthy metabolic profiles, including normal blood pressure, cholesterol levels, and blood sugar regulation [9]. In contrast, MUHO describes obese individuals who exhibit adverse metabolic markers such as insulin resistance, hypertension, or abnormal lipid levels, placing them at higher risk for CVD and other health issues [8]. In addition, normal-weight individuals who exhibit metabolic abnormalities similar to those seen in obesity are classified as “metabolically unhealthy normal weight” (MUNW) [10]. Conversely, those of normal weight who do not have any metabolic disorders are categorized as “metabolically healthy normal weight” (MHNW) [11–13].

According to studies, subjects with MHO had a lower risk of CVD, especially heart attacks and death, than those with MUHO [11, 14]. As compared to MHNW, MHO had a marginally higher CVD and overall mortality rate [15]. The majority of MHO patients do not have diabetes, dyslipidemia, or hypertension [9, 11, 13]. Lifestyle factors such as dietary habits, environmental factors, and genetic factors might affect metabolic phenotypes [16]. These factors could explain the transition from MHO to MUHO [17]. Moderate physical activity with a healthy diet is the best way to promote metabolic health. In addition, environmental pollutants, particularly persistent organic pollutants (POPs), may be linked to obesity and other health problems [18]. Although genetic predisposition may play a role in MHO, lifestyle factors, including physical activity and dietary intake, may also play an important role in its etiology [19]. There are inconsistent findings related to the different metabolic phenotypes of obesity, lifestyle, and environmental factors. These relationships have not been adequately explored through a comprehensive scoping review. This scoping review aims to synthesize evidence on how dietary patterns, genetic variations, and environmental factors influence metabolic phenotypes in obese and normal-weight individuals, providing insights to inform targeted interventions and policies to address the global obesity epidemic.

## 2. Methods

The Arksey and O'Malley methodological framework for scoping reviews, which was extended by Levac D, Colquhoun H, O'Brien KK and Daudt HM, van Mossel C, and Scott SJ, was used in the present scoping review [20–22]. According to Arksey and O'Malley's suggestion [20], we followed five steps, including identifying the research question (objective), identifying the relevant studies (search methods), screening and selecting studies for review, extracting data and charting, and summarizing and reporting the results. Our findings were reported using the Preferred Reporting Items for Systematic Reviews and Meta-Analyses Extension for Scoping Reviews (PRISMA-ScR) guidelines.

### 2.1. Research Objective

In the first step, we defined research questions and search strategies. We want to provide a comprehensive literature summary on the association between genetic, environmental, and nutritional factors with the metabolic phenotypes of obesity.

### 2.2. Search Strategy

We conducted a systematic search in three electronic databases, including the MEDLINE database (PubMed), the Web of Science, and Scopus, up to the beginning of April 2024. The search strategy includes relevant search terms, synonyms, and medical subject headings (MeSH), which were combined using Boolean operators (AND, OR, and NOT). The details of the key terms and search lines are described in Table S1. Moreover, hand searches of relevant publications and reference lists of eligible articles were performed to identify any additional sources.

### 2.3. Study Selection

Observational studies that assessed any genetic, environmental, and/or nutritional aspects of the metabolic phenotypes of obesity in the general population were selected. Only English-language and human studies, without restriction on race, age, gender, and publication date, were included. Editorials, reviews, commentaries, viewpoint publications, and conference abstracts were excluded. These criteria were established to prioritize primary research that provides empirical data and rigorous analysis, aiming to minimize bias and derive robust conclusions based on solid empirical evidence.

### 2.4. Search Outcome and Study Screening

**Figure 1:**
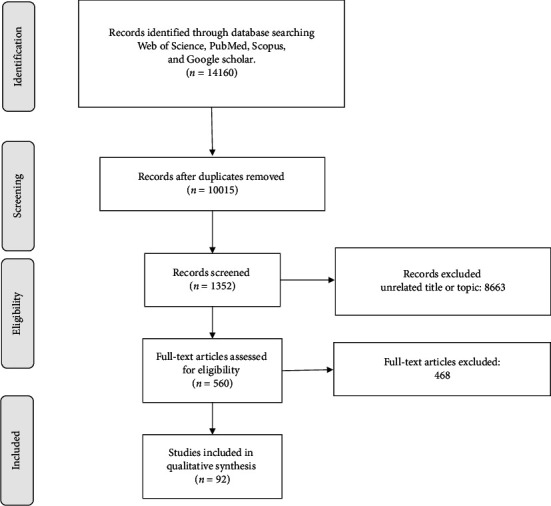
Flowchart of study selection for scoping review.

Two independent reviewers (MHB and RR) conducted a comprehensive literature search based on prespecified eligibility criteria in order to reduce selection bias. All identified records were exported to the EndNote reference manager, and duplicated records were removed. Two independent reviewers (HD and RR) screened the titles, abstracts, and full texts of the included articles. In addition, the references to related review articles were checked to find undetected, appropriate studies. Any discrepancy among the reviewers during the screening process was resolved using discussion in consultation with the third reviewer (RK). Details of the initial search outcome and screening process are shown in the PRISMA flowchart (Figure 1).

### 2.5. Data Extraction/Charting

The following items were extracted from suitable and relevant studies: first author name, publication date, study design, country of study, sample size, age of participants, exposure, exposure assessment tools, metabolic phenotypes of obesity definition, and main findings. The characteristics of the included studies are shown in Tables S2, S3, and S4.

## 3. Results

### 3.1. Description of Included Studies

A total of 14,160 original articles were identified. After the exclusion of the duplicated articles, the titles and abstracts of the remaining articles were screened. Finally, 92 relevant articles were included in this scoping review. 48 articles investigated the association between nutritional factors and metabolic phenotypes of obesity, 22 articles focused on environmental factors, and 22 articles assessed the genetic factors. The sample size of the studies varied between 20 and 205,745 samples, and the age range was between 4 and ≥ 80 years. A summary of the included studies is shown in Tables S2-S4.

In the present scoping review, each factor related to the metabolic phenotypes of obesity was classified as modifiable or nonmodifiable. Genetic and sociodemographic factors were classified as nonmodifiable factors and nutritional and behavioral factors were classified as modifiable factors.

### 3.2. Nonmodifiable Factors

#### 3.2.1. Genetic and Epigenetic

Twenty articles studied the association between genetic and epigenetic factors with the metabolic phenotypes of obesity. Details of the included studies are summarized in Table S2. Most studies focused on the MHO and the MUHO. Five studies were conducted among children and adolescents [23–27].

One cohort study on 3317 Chinese children and adolescents reported that single-nucleotide polymorphisms (SNPs) rs6773957 and rs4783244 at the adiponectin gene (ADIPOQ locus) and adiponectin receptor (CDH13) were associated with metabolic abnormalities [23]. The highest expression of the Tripartite Motif Containing 11 (TRIM11) gene and the lowest expression of the ADAMTS-like protein 2 (ADAMTSL2) gene in MHO children versus MUHO children were reported [24]. Another cohort study on children and adolescents showed that miRNA 34a, 122, and 192 were related to obesity-associated inflammatory markers, including tumor necrosis factor (TNF) α, interleukin-1 receptor antagonist (IL-1Ra), procalcitonin, and adiponectin [24]. A cohort study among 1475 children and adolescents identified a considerable association between the MUNW phenotype and SNPs at the CDK5 regulatory subunit associated protein 1 like 1 (CDKAL1) gene. This cohort study revealed that the interaction between environment and genetics was an important factor in predicting the metabolically obese, normal weight (MONW) phenotype among children [25].

Cross-sectional studies showed that downregulation and upregulation of microRNAs were different in MHO and MUHO children and adolescents [24, 28].

A cohort study on 49,915 obese and normal-weight subjects reported that the genes for lipoprotein lipase (LPL), apolipoprotein A-V (APOA5), and cholesteryl ester transfer protein (CETP) were associated with metabolically unhealthy (MUH) phenotypes (MUHPs) [29]. A Spanish cohort of 1051 individuals revealed that higher methylation levels in the CpG sites cg20707527 (*ZFPM2*) could have a protective impact against the progression to unstable MHO, while higher methylation levels in cg11445109 (*CYP2E1*) would enhance the progression to MUHO. CYP2E1 may contribute to oxidative stress by increasing its activity. It has been shown that CYP2E1 activity is higher in patients suffering from obesity due to MetS [30]. DNA methylation at 22 sites in several genes related to NF-kappa-B signaling was associated with a metabolically healthy Z-score (MHZ) [31].

In a Mexican cross-sectional study, the +45T >  G SNP was associated with reduced odds of the MUHP [32]. Fat mass and obesity-associated (FTO) rs9939609 variants were significantly different among metabolic phenotypes of obesity. The highest frequency of the obesity-risk allele AA of the *FTO* gene was observed in MUHO, MHO, MUNW, and MHNW, respectively [33]. A cross-sectional study revealed that genetic background is an important factor associated with MHO and MUO related to obesity. Also, this study identified 12 obesity-predisposing genetic variants correlated with a MUO [34].

Five SNPs, including rs17782313, rs571312, rs656710, rs2331841, and rs12970134 in the melanocortin-4 receptor (MC4R), were associated with a higher risk of MUHO among Chinese adults aged 18–83 years. The presence of the A allele of rs2331841 had a higher correlation with the risk of obesity and MUHO in comparison to other SNPs [35]. A study on Beijing children and adolescents showed that the interaction of KCNQ1-rs237897 and rs237892 variants with lifestyle factors was associated with the prediction of MHO status [26].

In a cross-sectional study on 60 Dutch subjects, it was identified that the co-expressed genes of IL1B and IL-6 were different between MHO and MUHO individuals [36]. The T45T genotype of the adiponectin gene was associated with a higher risk of metabolic disorder in patients with abdominal obesity. In addition, the G45G adiponectin genotype carriage was associated with the benign metabolic status [37, 38].

In a cohort study of 13,597 Chinese people, a genetic risk score was found to be positively associated with the MHO or MUHO phenotype [39]. The T allele of the adiponectin T45G polymorphism was identified as a factor associated with MUHO [40]. A cohort study showed that the FTO rs9939609 SNP has no significant impact on obesity-related metabolic traits [41].

A case-control study showed that SNPs including rs1421085, rs1558902, rs1121980, and rs8050136 in FTO genes were risk markers of the MUHO phenotype [42]. Another case-control study showed that the mRNA expression levels of TLR2, MyD88, and NFĸB were not significantly different between MUHO and MHO participants [43].

### 3.3. Sociodemographic Factors

#### 3.3.1. Ethnicity and Race

A cross-sectional study on 1777 adult subjects (aged ≥ 20 years) showed that MHO individuals had the non-Hispanic White ethnicity (Table S4) [44].

#### 3.3.2. Socioeconomic Status (SES)

Four studies investigated the relationship between socioeconomic factors, including household income, parental education, and living area, with metabolic health. A study of 3650 Korean children and adolescents found that MHNW subjects had significantly higher household income than MUNW subjects [45]. A cohort study on 1475 Chinese children and adolescents showed that lower levels of household income and parental education were significantly associated with higher odds of the MONW phenotype [25]. Living in rural areas and having low levels of education were related to higher odds of the MUHP in a cross-sectional study among the 19,640 Thai population [46]. Lower maternal education was a considerable factor for MHO Chinese children. This study showed that in Chinese children with highly educated mothers, the odds of MHO-IR increased by 59% (Table S4) [26].

### 3.4. Birth Weight

Two cross-sectional studies on children and adolescents reported that birth weight was related to MHO. A study among Chinese children and adolescents aged 6–18 years showed that with each 1-kg increase in birth weight, the odds of MHO increased by 40% [26]. Also, in another study on 117 French children aged 6–15 years, weight gain (0–2 years) and increased fetal growth were protective factors against the development of central obesity and insulin resistance, possibly indicating MHO [47].

### 3.5. Environmental Pollutant

In five studies, the association between the metabolic phenotypes of obesity and environmental pollutants was investigated. High levels of POPs in serum or plasma were identified as a risk factor for MUHO in two cross-sectional studies [48, 49] and one case-control study [50] (Table S4).

A cross-sectional study on 431 Norwegians reported that dioxin-like polychlorinated biphenyls (PCBs) in the higher quartile ( ≥ 75th) were associated with MetS [51]. Also, a case-control study among young adults, aged 18–26 years, showed that short-term exposure to ozone (O3) had a negative effect on metabolic health in obese individuals in comparison to normal-weight subjects [52]. A study on 1392 overweight and obese individuals assessed 23 urinary metals and metabolic phenotypes and showed urinary levels of zinc and the zinc–copper ratio were associated with an increased risk of an unhealthy metabolic phenotype (Table S4) [53].

### 3.6. Modifiable Domain

#### 3.6.1. Nutritional Factors

Details of the included studies about nutritional factors are summarized in Table S3.

#### 3.6.2. Dietary Pattern

Dietary patterns play a significant role in influencing metabolic phenotypes. Thirteen studies considered the association between dietary patterns and metabolic phenotypes. Eight studies [19, 54–60] focused on the healthy/unhealthy and Western dietary patterns. They reported that healthy food groups, including fruits, vegetables, and fish, played an important role in metabolic phenotypes. Unhealthy dietary patterns are characterized by energy-dense foods, refined and processed foods, carbohydrate-rich foods, red meat, savory and/or sweet snacks, sugars, high-fat dairy, high-energy drinks and beverages, soda and juice, and alcoholic drinks that were positively associated with MUHO and MHO. Two cross-sectional studies among Lebanese adults revealed that the traditional Lebanese dietary pattern, characterized by high consumption of whole grains (e.g., bulgur and freekeh), legumes (e.g., lentils and chickpeas), vegetables (e.g., leafy greens and tomatoes), fruits (e.g., citrus and figs), olive oil, fish, and poultry, with limited intake of processed meats, refined carbohydrates, and sugary beverages, was associated with higher odds of MHO [19, 61]. Four cross-sectional studies [62–65] and one cohort study [66] highlighted the protective role of the Mediterranean dietary pattern against MUHO. This pattern, characterized by high consumption of fresh fruits (e.g., grapes and olives), vegetables (e.g., spinach and eggplant), whole grains (e.g., barley and farro), legumes, nuts, seeds, fatty fish (e.g., sardines and mackerel), and olive oil, with moderate dairy (e.g., yogurt and cheese) and red wine intake, is linked to improved lipid profiles and reduced oxidative stress due to its high content of omega-3 fatty acids, polyphenols, and fiber [67]. Two studies investigated the relationship between adherence to the dietary approaches to stop hypertension (DASH) diet, as measured by a DASH score, and metabolic phenotypes of obesity. The DASH diet, characterized by high consumption of fruits, vegetables, whole grains, low-fat dairy, lean proteins, nuts, and seeds, with limited sodium, red meat, processed foods, and added sugars, was associated with lower odds of MUHO due to its high content of potassium, magnesium, and fiber [17, 68]. A cross-sectional study on 504 Iranian adults demonstrated that an animal dietary pattern (ADP) including red meat, organ meat, poultry, fish, nuts, and dry fruits was associated with MHO and MUHO [54]. Another cross-sectional study showed that obese/overweight individuals with a diet including tea, coffee, snacks, pizza, processed meat, red meat, organ meat, fish, refined grains, fruit juice, high-energy beverages, and tomatoes were more likely to have an unhealthy metabolic phenotype [69].

Specific food groups also play a role in metabolic health within these patterns. A cross-sectional study of 1047 Arabian adolescents showed that vegetable intake was positively associated with MHO [70]. Lower carbohydrate and adequate protein intake were related to the lower odds of MUNW. According to the results of a cohort study on Canadian children aged 8–10 years, a lower daily intake of vegetables and fruits was associated with the transition from MHO to MUHO [71]. According to the findings of cross-sectional studies [55, 56], higher intakes of vegetables and fiber were positively associated with MHO. Consumption of salty snacks was more common among MUHO subjects in a cross-sectional study among Iranian students [72]. Also, according to the results of a cohort study on Iranian adults, a higher intake of organ meats, fast foods, and potatoes increased the risk of developing a MUH status. This study showed that higher consumption of dairy products, poultry, apples/pears, citrus fruits, and tea or coffee reduced the risk of MUH status [73]. Coffee may protect against cardiometabolic risk by raising the metabolic rate and reducing energy intake. Further research is required to determine whether the potential negative effects of coffee on MetS components are dependent on how much coffee is consumed or on the additives consumed with coffee, such as sugar or milk.

The cross-sectional study showed that high consumption of fruit and vegetables was related to lower odds of MUHO. In addition, one to lower than 3 times consumption of seafood/fish in a month significantly reduced the risk of MUHO [55]. However, in a large cross-sectional study on 4855 American adults, a healthy dietary pattern including vegetables, fruits, 100% fruit juice, sugar-sweetened beverages (SSBs), whole grain bread, refined grains, beans, fish, poultry, red meat, processed meat, fried foods, low-fat dairy, and high-fat dairy was not associated with MHO [74–76]. Another large cross-sectional study on Caucasian subjects showed that fish intake was not different between MHO and MUHO subjects [77].

#### 3.6.3. Macronutrients and Food Groups

Macronutrient intake is crucial for maintaining metabolic health. In children, lower protein intake was linked to the transition from MHO to MUHO [71]. A cross-sectional study showed that carbohydrate, fat, and total energy intake in MUHO girls were significantly lower than in MHO girls [78, 79]. In addition, obese boys with a MUH status had a significantly higher intake of protein [78, 80].

A study of 299 overweight/obese nonshift worker adults showed that the risk of MUHO increased by consuming less amount of energy earlier in the day and more energy and carbohydrates later [81]. While in another cross-sectional study on 775 American adults, diet composition (including macro- and micronutrients) was not related to the metabolic phenotype [82].

Higher consumption of polyunsaturated fatty acids (PUFAs), especially n-6 PUFA, was observed among MUHO subjects in comparison to MHO and nonobese subjects [83]. In contrast, lower intakes of saturated fat were positively associated with MHO [55, 74, 77, 83].

#### 3.6.4. Micronutrients

In Brazilian adults aged 21–59, 25(OH)D deficiency was linked to MUHO [13]. A cross-sectional study on prepubertal Spanish children showed that overweight or obese children with MUH status had higher levels of ferritin and hemoglobin than others [84]. A study on 2201 American adults showed that high consumption of coffee among overweight subjects was associated with MUHP. This study showed that MUHP was positively associated with a high plasma folate level in both overweight and obese subjects [85]. Favorable effects of flavonoid intake on metabolic health were shown in a cohort study among the 1114 Iranian population. In this cohort, a higher intake of flavonoids and carotenoids, including flavonols, flavones, isoflavones, flavanones, and anthocyanins, was associated with a lower risk of MUHP [73]. In addition, a higher magnesium intake was associated with reduced risk of MUH status [79], and lower calcium intake was observed in MUHO girls compared to MHO girls [78, 79].

#### 3.6.5. Dietary Index

Higher dietary inflammatory index (DII) scores were a potential risk factor for MUHO. A high intake of proinflammatory foods such as red meat, refined carbohydrates, and saturated fat was associated with several metabolic alterations [86]. Another cross-sectional study among 1235 American adolescents (aged 12–18 years) and adults (aged 19–85 years) showed that obese individuals with better metabolic health status had higher diet quality based on the healthy eating index. This study found that obese adolescents with poor metabolic health consumed more servings of dairy and derived a lower percentage of their energy from solid fats and alcoholic beverages. Also, MHO females had higher consumption of whole grains, whole fruit, meat, and beans [56].

### 3.7. Lifestyle and Behavioral Factors

Twelve studies examined the relationship between behavioral factors, including physical activity, sedentary behaviors, sleep habits, smoking status, and alcohol consumption (Table S4).

#### 3.7.1. Physical Activity

Moderate-to-vigorous levels of physical activity were identified as a protective factor for metabolic health among obese subjects in most of the studies. While two studies showed that physical activity levels were not significantly different between MHNW and MUNW subjects (Table S4), increased physical activity, such as walking to school, plays an important role in improving general health status and maintaining metabolic health among obese and overweight subjects and even in normal-weight people (Table S4).

#### 3.7.2. Sedentary Behaviors

One cross-sectional study among 2195 American adults showed that time and level of sedentary activity, such as television viewing, were not significantly different between metabolic phenotypes of obesity [82], while another study showed that MHO women had lower sedentary time than MUHO women (Table S4) [77].

#### 3.7.3. Sleep

The relationship between sleep habits (such as sleep duration, sleep quality, and waking-up habits) and metabolic health status was studied in four cross-sectional studies, one cohort study, and one survey study [44, 45, 82, 87, 88]. In a study on the 1777 adult population, sleep duration was not significantly associated with MHO, but this study showed that regularly waking up during the night, feeling overly sleepy during the day, feeling unrested during the day, and having trouble falling asleep were associated with lower odds of MHO [44]. Other studies showed that sleep duration was significantly associated with the metabolic phenotype of obesity in both children and adults. These studies revealed that short sleep duration (mainly less than 6 h) was more common among MUHO subjects than MHO subjects [45, 80, 82, 87, 88]. A large cross-sectional study on 3650 children and adolescents showed that subjects with very short sleep duration (≤ 5 h) had a higher risk of MUNW and a lower risk of MHNW in comparison to subjects with normal sleep duration (8–10 h) [45]. Furthermore, subjects with a longer sleep duration (≥ 11 h) had a considerably higher risk of MUNW. While another large cross-sectional study in adults showed that long sleep duration (≥ 9 h) was less common in the MUHO group than in the MHO group (Table S4) [45].

#### 3.7.4. Smoking Status and Alcohol Consumption

Smoking status, whether current or former, was not significantly different between metabolic phenotypes of obesity in two large cross-sectional studies on 2195 American people and 2047 Irish people [82, 89]. However, in another study, MHO subjects were never smokers [44]. Also, former smoking was associated with lower odds of MUHO among the total and overweight population in a cross-sectional study of 2287 adults [55]. Another large cross-sectional study on 19,640 Thai adults showed that one of the characteristics associated with MUHO was current tobacco smoking [46].

Alcohol consumption was not correlated with metabolic health in two studies on the Irish and Thai populations [46, 89]. Whereas, a recent study showed that low intake of alcohol was associated with lower odds of a MUHP in overweight subjects (Table S4) [55].

### 3.8. Psychological Factors

#### 3.8.1. Job Stress

We found only one study that focused on the association between job stress and metabolic health phenotypes. In this study, the low skill discretion was associated with adverse metabolic health, that is, a lower score of skill discretion increased the odds of MUHO by 52%, while this study did not find a significant association between job stress and metabolic health in obese individuals (Table S4) [90].

## 4. Discussion

This scoping review assessed findings that investigated the associations between genetics, nutrition, physical activity, sedentary behaviors, sleep duration, smoking status, alcohol consumption, and psychological factors with obesity metabolic phenotypes.

In order to reduce the incidence of metabolic disorders related to obesity, genetic analysis may be useful for screening high-risk individuals. According to the genomewide association studies (GWAS), 38 variants were associated with phenotypes of obesity. The present study elucidates whether some of the variants are associated with metabolic phenotypes. The findings of five studies among children and adults showed that gene expression of TRIM11, ADAMTSL2, TNFα, IL-1Ra, procalcitonin, adiponectin, and CDK5 significantly contributed to MUHPs starting at a young age and lasting into adulthood [23, 25, 27, 32].

The genetic studies by Yang SS, Wang Y, He Y, Xu L, Jin Y, Zhang WS, Jiang CQ, Cheng KK, and Lam TH highlighted the genetic risk factors for increasing BMI and also appeared to be associated with the development of a new MUHO status [39]. A higher MUHO status is associated with inflammatory diseases [91]. MUHO is directly associated with greater levels of T2DM, CVD, and even death in comparison to MHO status [92]. The NOD-like receptors (NLRs) are important proteins that detect cellular stress, and their activation triggers the formation of the inflammasome, which produces IL-1β and IL-18 [93]. A study found that MHO individuals have greater NLRP3 inflammasome activity than MUHO individuals [94].

To our knowledge, the first study to identify genetic loci linked to MHO found that KCNQ1-rs2237892 and rs22237897 have a strong association with MHO [26]. According to GWAS in Asian and European populations and the Chinese population, the association of KCNQ1-rs2237897 and KCNQ1-rs2237892 with MHO has been confirmed [95–98]. In addition, KCNQ1-rs2237892 and rs22237897 are associated with MUHO based on insulin resistance or cardiometabolic risk factors [26]. One of the important roles of KCNQ1 is to encode the pore-forming subunit of a voltage-gated K+ channel that is predominantly expressed in pancreatic islets. KCNQ1 is responsible for activating and maintaining insulin secretion in response to glucose [99].

Some SNPs in the FTO gene were associated with the metabolic phenotype of obesity [41, 100–108]. SNPs rs6773957 at the adiponectin gene (ADIPOQ locus) and rs4783244 at the adiponectin receptor CDH13 were linked to metabolic abnormalities independent of BMI. Adiponectin levels were found to be associated with genetic variations. These loci were found to be more closely linked to metabolic health in children of normal weight than in those who were overweight or obese. Adiponectin-related genetic variants were more correlated with metabolic health in normal-weight subjects than in overweight/obese subjects. So, adipose tissue dysfunction, including alteration in adipokine secretion, is more important for metabolic abnormalities than increased fat mass alone [23]. Studies demonstrated that abnormalities in microRNA expression, including miRNA 34a, 122, and 192, were associated with unhealthy phenotypes and obesity-associated inflammation markers such as TNFα, IL-1Ra, adiponectin, and procalcitonin [27, 109–112]. It was shown that miRNAs are very stable in blood and therefore provide the best possibility for use as biomarkers.

BMI may be uniquely correlated with CpG sites in individuals with metabolic health and those with obesity. Several studies have suggested a link between DNA methylation and metabolic health about BMI [31, 113].

The assessment of methylation patterns may offer therapeutic targets for the prevention of metabolic deterioration in subjects with MHO. It is unknown how ADIPOQ and PPARG genetic variants influence the MUHP and metabolically healthy phenotype in subjects with either excess body weight or normal body weight [32]. There was no significant difference in the prevalence of the +276G > T ADIPOQ polymorphism between the MHO and MUHO phenotypes [37]. In addition, MUHO subjects with abdominal obesity were more likely to carry the +45T ADIPOQ allele than MHO adults. In addition, there was an association between SNP +45T > G and hypertriglyceridemia in subjects with abdominal obesity who had the genotype TT and also had an increased risk of metabolic disorders [32, 37].

Twelve obesity-predisposing genetic variants were found to be associated with a MUHO [34], which regulates physiological processes, including food intake (LEP), circadian rhythm (CLOCK), signaling at the synapses (CNR2 and BDNF), secretion of bile (ABCB11), expenditure of energy (UCP3), metabolism of lipids (PLIN1 and PPARGC1A), production of hormones (GNAS), reward-personalization genes (ANKK1), and insulin signaling processing genes [114, 115].

Some key factors, including age, maleness, percent body fat (PBF), the polymorphism T45G of adiponectin, and leptin, are associated with the progression from MHO to MUHO. Hyperleptinemia and leptin resistance play a key role in the pathogenesis of MUHO. So, MUHO may increase the risk of other chronic diseases through hyperleptinemia [116]. Comprehensive analyses also revealed that changes in adiponectin oligomer ratio and blood glucose levels may contribute to the effects of the T45G polymorphism on metabolic phenotypes [40]; the major differences between MUHO and MHO are related to the polymorphism of the adiponectin T45G chain. Adiponectin T45G polymorphism is associated with higher glucose levels and lower high molecular weight (HMW)/low molecular weight (LMW) ratios [30].

Genetic and epigenetic factors produce substantial interindividual variability that may be most comprehensively observed throughout the life cycle and may be considered precursors of later-onset diseases that begin in childhood [117].

In addition to genetic markers, lifestyle (calorie intake, dietary pattern, and physical activity) has a strong impact on the relationship between high-risk alleles and overweight or obesity. Thus, obesity can be prevented by lifestyle changes in people with high-risk alleles.

Healthy dietary patterns, including traditional or Mediterranean dietary patterns or the DASH diet, are closely associated with metabolic health in both obese and normal-weight individuals.

Many mechanisms have been implicated in the beneficial effects of the Mediterranean diet on cardiometabolic health, but improvements in insulin sensitivity, blood pressure, endothelial function, lipid profile, and biomarkers of inflammation are the most likely reasons [118–122].

The deficiency of some nutrients, including vitamin D, vitamin B9, flavonoids, and carotenoids, is positively associated with the development of MUHO [13, 73, 123]. Intake of flavonoids in both normal-weight and obese adults is associated with a lower MUHP. Higher intakes of total flavonoids, because of their antioxidant, antiobesity, and antidiabetes properties, may potentially reduce the severity of hypertriglyceridemia and MetS [123, 124]. Vitamin D and magnesium impact insulin activity, insulin production, blood pressure regulation, lipid profiles, and MetS [125]. In some MUHO individuals, vitamin D supplementation may play a role in reducing the incidence and aggravation of health problems associated with obesity [13].

Unhealthy dietary patterns such as western, animal, high-fat, and proinflammatory dietary patterns (IDPs) are positively associated with MUHO and increase obesity-risk factors [54, 57, 58, 69, 86, 126]. High concentrations of fats, sugars [127], transgreasy acids, saturated fats, and cholesterol in the western dietary pattern could lead to MUHO [128, 129]. The association between MUHO and a high intake of proinflammatory foods depends on the total energy intake. Some evidence shows that gene–environment interactions may partially explain the association between dietary patterns and cardiometabolic phenotype [54].

Individuals with normal weight and those who are overweight are both affected by inflammation in the pathophysiology of metabolic abnormalities [69, 89]. MHO has been speculated to be a transient phenotype [130], and it may be at greater risk for developing cardiometabolic disorders and converting to MUHO. There is an inverse relationship between the DII and cardiometabolic risk factors [131]. These results may be caused by the negative correlation of caffeine, tea, and fruit juices, as well as green and dark yellow vegetables, with IDPs [132].

The DASH diet is rich in nutrients such as potassium, magnesium, and vitamin C, as well as a variety of phytochemicals, which may decrease the risk of obesity, insulin resistance, and MetS [133–135]. Vegetables and fruits are the major food groups for healthy dietary patterns, which are associated with improved metabolic health and are also associated with better nutritional habits. It has been shown that consumption of foods belonging to these categories is inversely correlated with inflammation biomarker measures such as high-sensitivity C-reactive protein (hs-CRP) [136]. Also, some studies have reported that micronutrient and macronutrient intake and healthy food groups, including fruits and vegetables, did not have a significant association with metabolic phenotypes, even in individuals with normal weights, as well as those who are overweight [73]. Studies define metabolic health, or MHO, in different ways, which leads to discrepancies in results. It is important to agree on a definition of metabolically healthy phenotypes and develop dietary interventions based on the definition in order to create appropriate dietary strategies.

Protein intake can be associated with metabolic health. However, there are various findings related to sources of dietary protein and metabolic phenotypes [61, 137, 138].

A combination of fiber and mono- and polyunsaturated fats can increase insulin sensitivity, enhance fat oxidation, and reduce cardiovascular risk through their colonic, intrinsic, and hormonal effects. A diet that contains legumes, fruits, and vegetables has numerous phytochemicals, antioxidants, and fiber that may decrease oxidative stress and inflammation and enhance insulin sensitivity [61].

There may also be significant variations in relationships between diet and metabolic phenotypes according to gender among adults [57]. Physiological differences between females and males and differences in diet reporting could account for certain sex-based differences in dietary intake [54].

Some factors related to SES and the environment are associated with metabolic health problems. Job stress and exposure to organic pollutants lead to the development of MUHO.

Some studies showed that people who increase physical activity at any weight and gender have a lower risk of developing an unhealthy metabolic phenotype [80, 139]. The results of studies regarding the amount of physical activity on metabolic phenotypes are different. There is a need for further research to determine how much physical activity is needed for each metabolic health phenotype [140, 141]. Even without weight loss, increasing physical activity may improve the adiposity profile and metabolic risk [142]. Additionally, the MHO individuals engaged in more physical activity than the MUHO [143–145]. A significant relationship between physical activity and MHO was found between moderate physical activity in women and vigorous physical activity in men [57].

It has been shown that people with normal weight and obesity who have regular and adequate sleeping habits have a lower probability of having unhealthy metabolic phenotypes. An analysis of a Chinese study showed that adults who sleep longer than 9 h were more susceptible to being classified as MHO groups [45]. Also, both longer and shorter sleep durations were related to metabolic phenotypes [88, 146, 147]. In addition, research has suggested that sleep duration plays a role in the development of cardiometabolic risk factors [88], and more studies are needed to explain the association between sleep habits and metabolic abnormalities. Short sleep duration (< 6 h) may increase sympathetic activity, cortisol levels, and circadian disruption, impairing glucose metabolism and promoting insulin resistance, contributing to MUHO and MUNW phenotypes [88, 146, 148]. It may also elevate ghrelin and suppress leptin, increasing energy intake and fat accumulation [149]. Conversely, long sleep duration (≥ 9 h) may reflect inflammatory or neuroendocrine dysregulation, predisposing individuals to MUHO [45, 147, 150]. Optimal sleep duration is crucial for metabolic health and preventing MHO progression to MUHO.

The COVID-19 pandemic disrupted lifestyle factors critical to metabolic health, potentially increasing the prevalence of MUHO. Lockdowns reduced physical activity due to limited access to recreational spaces, increasing sedentary behaviors linked to insulin resistance [151, 152]. Dietary shifts toward processed foods and reduced fruit and vegetable intake, driven by supply chain issues and stress, likely promoted MUHO [153, 154]. These disruptions may have accelerated the transition from MHO to MUHO, requiring further research into long-term impacts.

Results related to smoking habits and alcohol consumption are inconsistent. [82]. Smoking or alcohol consumption may elevate triglyceride levels, blood pressure, and blood glucose and decrease HDL-C levels. Maintaining a healthy weight is an effective way to control insulin resistance, high triglycerides, and low HDL-C in alcohol consumers and smokers [155].

Genetic variants, such as FTO rs9939609 and adiponectin T45G polymorphisms, show stronger associations with MUHO in females, likely due to hormonal effects on fat distribution and insulin sensitivity [33, 37]. In terms of nutrition, Iranian girls with MUHO consumed less carbohydrate and fat compared to MHO girls, while MUHO boys had higher protein intake, suggesting sex-specific dietary impacts [72, 73]. In addition, MHO females consumed more whole grains and fruits, indicating that diet quality may influence females more significantly [56]. Environmental and lifestyle factors also vary by sex; moderate physical activity was linked to MHO in females, whereas vigorous activity benefited males [57]. The short sleep duration (< 6 h) showed a stronger association with MUHO in females, possibly due to greater sensitivity to sleep-related metabolic disruptions [45]. Socioeconomically, lower maternal education increased the odds of MHO in girls, reflecting potential gender-specific cultural or behavioral influences [26]. These findings underscore the need for sex-specific interventions, though the limited availability of sex-stratified data highlights the necessity for further research.

The association between birth weight and metabolic phenotypes is mediated by biological mechanisms originating in early life that shape long-term metabolic health. Low birth weight (LBW), often due to intrauterine growth restriction, may induce epigenetic changes (e.g., IGF2 and PPARG methylation), impairing glucose metabolism and increasing risks of MUHPs (MUHO/MUNW) [156, 157]. High birth weight (HBW), linked to maternal overnutrition, may cause excessive fetal adiposity and β-cell dysfunction, promoting MUHO via insulin resistance [158]. Rapid postnatal weight gain increases visceral fat and proinflammatory adipokines (e.g., TNF-α), driving MHO-to-MUHO transitions [47, 159]. Postnatal diets may exacerbate or mitigate these risks [78]. Thus, LBW and HBW contribute to metabolic phenotypes through distinct pathways.

This study included only observational studies, limiting the ability to establish causal relationships between genetic, environmental, and nutritional factors and metabolic phenotypes. In addition, it did not systematically assess the quality or risk of bias in the included studies, which may affect the reliability of the findings. Although the search strategy encompassed individual foods and nutrients, the emphasis on dietary patterns in many studies may have underrepresented the role of specific foods in shaping metabolic phenotypes. Furthermore, restricting the review to English-language studies may introduce language bias, potentially excluding non-English studies that could provide valuable insights into these factors across diverse populations.

## 5. Conclusion

This scoping review highlights the role of genetic, environmental, and nutritional factors related to the metabolic phenotypes of obesity. Genetic predispositions, such as ADIPOQ, FTO, and KCNQ1 polymorphisms, highlight the clinical need for genetic screening to identify high-risk individuals, enabling personalized interventions such as anti-inflammatory dietary plans for those with proinflammatory genetic profiles. Public health strategies should address MUHPs (MUHO/MUNW) by targeting modifiable factors, including unhealthy diets (e.g., high processed foods and saturated fats), physical inactivity, improper sleep habits, and environmental pollutants such as POPs. Early-life interventions targeting maternal health and infant growth patterns may disrupt pathways leading to MUHPs, offering a preventive strategy against obesity-related comorbidities. These findings support categorizing individuals by metabolic phenotype to predict disease risk and develop targeted strategies to improve metabolic health and reduce obesity-related diseases, such as T2DM and CVD.

## Data Availability

The data that support the findings of this study are available from the corresponding authors upon reasonable request.

## Supporting Information

Supporting Information Supporting Table 1: Details of the key terms and search lines.

Supporting Information Supporting Table-2: Characteristics of the included studies about genetic and epigenetic.

Supporting Information Supporting Table-3: Characteristics of the included studies about nutritional factors.

Supporting Information Supporting Table-4: Characteristics of the included studies about environmental factors.
