# Supplementary material for: Association of Genetic, Environmental, and Nutritional Factors With Metabolic Phenotypes of Obesity: A Scoping Review
Source: J Obes. 2025 Jul 2;2025:8472196. doi: 10.1155/jobe/8472196 (PMC12259317; doi:10.1155/jobe/8472196)
Supplement: Supporting Information — Supporting Table 1: Details of the key terms and search lines. [file 8472196.f1.docx]

Table S1- search strategy used in this scoping review

| **Factor** | **Search terms** |
| --- | --- |
| **Nutritional** | ("metabolically healthy" OR "metabolically abnormal" OR "metabolically unhealthy" OR "metabolic phenotype" OR "metabolically healthy non obese*" OR" metabolically healthy obese*" OR "metabolically non healthy non obese*" OR "metabolically non healthy obese*") AND (diet* OR nutrition OR "nutritional status" OR "dietary pattern"). |
| **Genetic** | ("metabolically healthy" OR "metabolically abnormal" OR "metabolically unhealthy" OR "metabolic phenotype" OR "metabolically healthy non obese*" OR" metabolically healthy obese*" OR "metabolically non healthy non obese*" OR "metabolically non healthy obese*") AND (gene* OR " genotype-phenotype associations­" OR " genotype-phenotype correlation­" OR "epigene*­"). |
| **Environmental** | ("metabolically healthy" OR "metabolically abnormal" OR "metabolically unhealthy" OR "metabolic phenotype" OR "metabolically healthy non obese*" OR" metabolically healthy obese*" OR "metabolically non healthy non obese*" OR "metabolically non healthy obese*") AND  (Environment* OR “air pollution” OR climate OR “ultraviolet radiation” OR “green space” OR “industrial pollution” OR “water quality” OR “noise pollution” OR “low-frequency” OR “radiofrequency radiation” OR radon OR “nuclear facilities” OR contamination OR “physical activity” OR sleep OR “socioeconomic factor*” OR race OR ethnicity OR "food environment*­" OR "chemical environment*­" OR "social environment*­" OR "home environment*­" OR "gene environment interaction" OR "" OR "built environment" OR "physical environment" OR "work environment" OR "natural environment" OR "transportation"). |
