# Supplementary material for: Association of Genetic, Environmental, and Nutritional Factors With Metabolic Phenotypes of Obesity: A Scoping Review
Source: J Obes. 2025 Jul 2;2025:8472196. doi: 10.1155/jobe/8472196 (PMC12259317; doi:10.1155/jobe/8472196)
Supplement: Supporting Information — Supporting Table-2: Characteristics of the included studies about genetic and epigenetic. [file 8472196.f2.docx]

Table S2- characteristics of included studies about genetic and epigenetic.

| **ID** | **Author**  **(year)** | **Study design** | **Sample size**  **(age/ race)** | **Genetic factor** | **Obesity phenotype and definition and metabolic criteria** | **Main finding** |
| --- | --- | --- | --- | --- | --- | --- |
| 1 | ^(116)^Han, F et al. (2024) | Case-control | 300  (Chinese adult participants) | 18 single nucleotide polymorphisms (SNPs) and 31 CpG  sites | metabolically healthy obesity (MHO) cases and metabolically healthy normal-weight (MHNW) | The study showed that the rs4714211 polymorphism of the GLP1R gene was strongly related with MHO. GLP1R methylation sites (GLP1R−68-CpG 7.8.9; GLP1R−68-CpG 12.13; GLP1R−68-CpG 17; GLP1R−68-CpG 21) were linked to MHO. Two of these methylation sites (GLP1R−68-CpG 7.8.9; GLP1R−68-CpG 17) partially influenced the link between genotypes and MHO. |
| 2 | ^(39)^Rasaei N, et al. (2023) | Cross-sectional | 279  (women with overweight and obesity) | melanocortin-4 receptor (MC4R) gene | metabolically healthy obese (MHO) and metabolically unhealthy obese (MUO) | Participants with CC genotype is more likely to have an unhealthy phenotype with an increase in N6/N3 as one fat quality indices than those who do not have CC genotype. The study found the interaction of dietary fat quality indices such as N6/N3 and the MC4R gene in metabolically unhealthy overweight and obese women. |
| 3 | ^(24)^Li G, et al. (2022) | Cohort | 3,317  (6–18 years old/ Chinese children) | rs6773957 at the adiponectin gene (ADIPOQ locus) and rs4783244 at the adiponectin receptor CDH13 | Metabolic unhealthy (MUH) | The study found that SNPs rs6773957 at the adiponectin gene (ADIPOQ locus) and rs4783244 at the adiponectin receptor CDH13 were correlated with metabolic abnormalities, independent of BMI at baseline. |
| 4 | ^(29)^Rovira-Llopis S, et al.  (2021) | Cross-sectional | 20  (18-70 years old/ Spanish population) | miRNA | metabolically healthy obese (MHO)  metabolically unhealthy obese (MUO) | The study shows a pattern of differentially expressed miRNAs in obesity according to MetS, and identify those related to insulin resistance and lipid metabolism pathways. A remarkable alteration was observed in the biochemical metabolic profile of MUHO population, and this profile was accompanied by substantial differences in the pattern of miRNA expression, including 72 upregulated and 87 downregulated miRNAs in the serum of MUHO vs. MHO subjects. |
| 5 | ^(25)^Plaza-Florido A, et al.  (2020) | Cross-sectional | 27  (10.1 ± 1.3 years old/ Spanish children) | TRIM11  ADAMTSL2 |  | The study identified 32 genes differentially expressed in children with MHO compared to MUHO, which were related to metabolism, mitochondrial, and immune functions. These findings provide understanding the underlying mechanisms that differentiate MHO from MUHO in young ages. This study showed the highest expression of TRIM11 gene and the lowest expression of the ADAMTSL2 gene in children with MHO compared to MUHO. |
| 6 | ^(30)^Park J, et al. (2021) | Cohort | 49,915  (40–79 years' old  /Korean population) | LPL  APOA5  CETP | (1) MHNW (metabolically healthy normal weight)  (2) MUHNW (metabolically unhealthy normal weight)  (3) MHO (metabolically healthy obese)  (4) MUHO (metabolically unhealthy obese) | The study found that LPL, APOA5, and CETP were associated with metabolically unhealthy phenotypes among both normal weight and obese individuals. |
| 7 | ^(28)^Lischka J, et al. 2021 | Cohort | 109  (9-19 years old/ Austrians children) | miRNA 34a, 122, and 192 | metabolically healthy obese (MHO)  metabolically unhealthy obese (MUO) | The study exhibited MicroRNAs (miRNAs) patterns associated with obesity-associated inflammation and also revealed miRNAs elevated in metabolic disorders in youth with obesity. Among those, the study showed for the first time that miRNA 34a, 122, and 192 were additionally linked to obesity-associated inflammatory markers TNFα, IL-1Ra, adiponectin, and procalcitonin. |
| 8 | ^(31)^Gutiérrez-Repiso C, et al. 2021 | Cohort | 1051  (18–65 years old/ Pizarra population) | ZFPM2  CYP2E1 | metabolically healthy obese (MHO) | DNA methylation status is associated with the stability/worsening of as MHO phenotype. Two potential biomarkers of the transition to an unhealthy state were identified and deserve further investigation (cg20707527 and cg11445109). In addition, the described differences in methylation could alter immune system-related pathways, highlighting these pathways as therapeutic targets to prevent metabolic deterioration in as MHO patients. |
| 9 | ^(32)^Do W, et al. 2021 | Cohort | 7497  (45–64 years old/ Chinese American people) | cg18989722 | metabolic syndrome  (MetS ) | Four CpG sites which may have a unique relationship with BMI in metabolically healthy vs unhealthy individuals. Differential associations between DNAm and BMI by MHZ were identified at 22 sites, one of which was validated (cg18989722) and three of which were predictive of incident CHD. These sites are located in several genes related to NF-Kappa-B signaling, suggesting a potential role for inflammation between DNA methylation and BMI associated metabolic health. |
| 10 | ^(33)^Torres-Castillo N, et al. 2020 | Cross-sectional | 345  (18–65 years old/ Western Mexican adults) | +45T>G ADIPOQ | normal weight metabolically healthy (NWMH)  normal weight metabolically unhealthy (NW-MUH)  excess weight metabolically healthy (EW-MH)  excess weight metabolically unhealthy EW-MUH. | Lower serum adiponectin levels were associated with the metabolically unhealthy (MUH) phenotype in excess weight (EW) subjects. In addition, the +45T>G SNP was associated with reduced odds of the MUH phenotype. There were no differences in frequencies between ADIPOQ genetic variants and phenotypes; however, SNP –11377C>G and carriers of two copies of the GG haplotype of ADIPOQ (–11391G>A and –11377C>G) displayed lower serum adiponectin concentrations in the entire population. |
| 11 | ^(43)^Sedaghati‑khayat B, et al.  (2018) | Case-control | 954  (43 ± 16 years old/ Iranian adults) | FTO | metabolic syndrome  (MetS ) | This study showed that four markers (rs1421085, rs1558902, rs1121980 and rs8050136) in the first intron of the FTO gene should be the risk marker in MUHO participants. |
| 12 | ^(26)^ Li G, et al. 2020 | Cohort | 1475  (6–18 years old/ Chinese children) | CDKAL1 rs2206734 | metabolically obese normal weight (MONW) | The study revealed the novel evidence that early environment (especially birthweight) and genetics, along with their interaction with one another, play important roles in predicting the MUNW phenotype among children. Indeed, this study identified an association in this population between the MUNW phenotype and SNPs at the obesity susceptibility locus (CDKAL1) within the gene encoding cyclin dependent kinase 5 (CDK5) regulatory subunit-associated protein 1-like 1. Moreover, the protective effects of the CDKAL1-rs2206734 gene variant could be strengthened by a favorable intrauterine nutritional environment and amplified further by the combined impact of protective childhood environmental factors like physical activity, fruit consumption, advanced parental education and high household income. |
| 13 | ^(34)^Abolnezhadian F, et al. (2020) | Cross-sectional | 165  (20- 65 years old/ Iranian adults) | FTO-rs9939609 | 1) MHNW: metabolically healthy normal weight  2) MUNW: metabolic  unhealthy normal weight  3) MHO: metabolically healthy obese  4) MUHO: metabolic unhealthy obese. | Results showed a significant difference in the FTO-rs9939609 variant in obesity phenotypes. The genotype frequencies of high-risk genotype (AA) were more in MUHO, MHO, MUNW and MHNW, respectively. Thus, FTO gene polymorphism is more closely associated with obesity than metabolically health. |
| 14 | ^(40)^Yang S, et al. (2019) | Cohort | 13,597  (at least 50 years old/Chinese population) | genetic risk score (GRS) | metabolically healthy obese (MHO)  metabolically unhealthy obese (MUO) | Study showed a positive association between genetic risk score (GRS) and the incidence of being MHO/MUO. |
| 15 | ^(35)^Ramos-Lopez O, et al.  (2019) | Cross-sectional | 298 participants  (Age of MHP group: 42.4 ± 10.3 years old and Age of MUP: 48.0 ± 9.9 years old/ Spanish adults) | rs7799039 (LEP)  rs4731426 (LEP)  rs1801260(CLOCK)  rs3123554 (CNR2)  rs569805 (ABCB11)  rs6265 (BDNF)  rs1685325 (UCP3)  rs1052700 (PLIN1)  rs8192678 (PPARGC1A)  rs6123837 (GNAS)  rs1800497 (ANKK1)  rs2860323 (TMEM18) | metabolically healthy phenotypes (MHP)  metabolically unhealthy phenotypes (MUHP) | The genetic background is an important factor explaining metabolically healthy phenotypes (MHP) and metabolically unhealthy phenotypes (MUHP) related to obesity, in addition to lifestyle variables.  12 obesity-predisposing genetic variants were associated with a MUP, which regulate physiological processes such as food intake (LEP), circadian cycle (CLOCK), neuronal synapse signaling (CNR2, BDNF), bile secretion (ABCB11), energy expenditure (UCP3), lipid metabolism (PLIN1, PPARGC1A), hormone production (GNAS), food rewarding (ANKK1), and insulin signaling (TMEM18). Furthermore, a weighted genetic-risk score (wGRS) constructed of the aforementioned obesity-risk alleles was strongly associated with a MUP and was the major contributor to this phenotype. |
| 16 | ^(36)^Gao L, et al. (2019) | Cross-sectional | 1100  (18–83 years old/ Chinese adults) | rs2331841  rs656710, rs17782313, rs571312, and rs12970134 (MC4R) | metabolically unhealthy obesity (MUHO (  metabolic unhealthy normal weight (MUH-NW (  metabolic healthy normal weight (MH-NW( | Participants with the A risk allele of rs2331841 had a higher risk of MUHO than other participants. Participants with low-frequency alleles of rs656710, rs17782313, rs571312, and rs12970134 (MC4R) had a higher risk of MUHO than other participants. |
| 17 | ^(44)^Amiri P, et al. (2019) | Case-control | 70  (18–60 years old/ Iranian adults) | TLR2  MyD88  NFĸB  IL-1β  FFAs | metabolically healthy obesity (MHO)  metabolically healthy obesity / )MUHO(. | Findings revealed that the expression levels of TLR2, MyD88, and NFĸB genes as well as IL-1β and FFAs were similar between MUHO and MHO groups. Moreover, when the two groups were matched on abdominal fat, it looks that gene expression levels of TLR2, MyD88 and NFĸB are highly related to abdominal obesity than to healthy or unhealthy metabolic state. |
| 18 | ^(41)^Chang CS, et al.  (2018) | Cross-sectional | 234  (103 males and 131 females (18—50 years old)/ Taiwanese people) | T allele of adiponectin T45G polymorphism, leptin, and percent body fat (PBF) | metabolically healthy obesity / )MUHO(. | The factors associated with MUHO are age, male gender, the T allele of adiponectin T45G polymorphism, leptin, and percent body fat (PBF). The net effects of T45G polymorphism on the MUHO phenotype may be achieved by changes in the adiponectin oligomerization and glucose levels. |
| 19 | ^(27)^ Li L, et al. (2016) | Cross-sectional | 1,213  (6–18 years old/ Beijing children) | KCNQ1-rs237897 and rs237892, | MHO-CR / MUO-CR | Study showed that common variants such as KCNQ1-rs237897 and rs237892, interacting with modifiable lifestyle factors, are associated with MHO, which suggests there is a partly genetic– environmental basis to the widely reported MHO phenotype. |
| 20 | ^(37)^Kogelman LJ, et al.  (2016) | Cross-sectional | 60  (44.85 ± 10.38 years old/ Netherlands people) | IL1B  IL-6 | metabolically healthy obesity (MHO) | Study showed that two altered genes between the MHO and MUHO subnetwork: IL1B and IL-6. Both genes showed, most of them significant, stronger inter-tissue co-expression in the MUHO subnetwork than in the MHO subnetwork. Even though IL-6 and IL1B mRNA levels were not altered between MHO and MUHO individuals in our study, their co-expression with other genes indicates a potentially important role in obesity-induced development of metabolic disturbances. |
| 21 | ^(38)^Berezina A, et al. (2015) | Cohort | 503  (30-55 years old/ Russian people) | G45G adiponectin  T45T adiponectin | metabolically healthy obesity (MHO)  metabolically unhealthy obesity. (MUHO) | Benign metabolic status was associated with higher physical activity, shorter duration of obesity, and G45G adiponectin genotype carriage. Also, In metabolically healthy individuals with abdominal obesity low frequency of T45T adiponectin gene polymorphism was found, which is associated with an increased risk of metabolic syndrome in patients with abdominal obesity. |
| 22 | ^(42)^Hsiao TJ, et al. (2016) | Cohort | 803  (30–75 years old/ Taiwanese population) | FTO rs9939609 | Obesity | Results show that FTO rs9939609 was associated with obesity risk in the Taiwanese participants. While, the study observed that FTO rs9939609 had no association with obesity-related metabolic traits. |
