# Supplementary material for: Association of Genetic, Environmental, and Nutritional Factors With Metabolic Phenotypes of Obesity: A Scoping Review
Source: J Obes. 2025 Jul 2;2025:8472196. doi: 10.1155/jobe/8472196 (PMC12259317; doi:10.1155/jobe/8472196)
Supplement: Supporting Information — Supporting Table-3: Characteristics of the included studies about nutritional factors. [file 8472196.f3.docx]

Table S3- characteristics of included studies about nutritional factors

| **ID** | **Author**  **(year)** | **Study design** | **Sample size**  **(age/ race)** | **Nutrition factor** | **Method of assessment of nutrition factor** | **Obesity phenotype and definition** | **Main findings** |
| --- | --- | --- | --- | --- | --- | --- | --- |
| 1 | ^(18)^Rahimi H, et al.  (2020) | Cross-sectional | 341  (Iranian children and adolescents with 6-13 years old) | Dietary Approaches to Stop Hypertension (DASH) score. | Valid and reliable food frequency questionnaire (FFQ). | metabolically healthy obesity (MHO)  metabolically healthy obesity / )MUHO(. | Participants in the highest tertile of the DASH score had significantly decreased odds for MUHO based on HOMA-IR compared with those in the lowest tertile, after adjustment for confounders. |
| 2 | ^(59)^Mirzababaei A, et al.  (2019) | Cross-sectional | 290  (Overweight and obese females aged 18-50 years old/ Iranian people) | Healthy dietary pattern (HDP)  Western dietary pattern (WDP)  unhealthy dietary pattern (UNHDP) | Valid 147 items semi-quantitative food frequency questionnaire (FFQ). |  | The dietary pattern and MUHO phenotypes were associated. Association between major dietary patterns and the metabolically healthy obesity (MUH) phenotype is new. A positive relationship was found between a WDP and unhealthy dietary pattern (UNHDP) with odds of the MUHO phenotype. |
| 3 | ^(55)^Nikniaz L, et al.  (2019) | Cross-sectional | 504  (Adults/East-Azerbaijan-Iran) | Animal dietary pattern(ADP)  Healthy dietary pattern(HDP)  .  Western dietary pattern (WDP) | 80-item quantitative FFQ. | 1.MHL (metabolically healthy lean):  2.MUHL(metabolically unhealthy lean):  3. MHO (Metabolically healthy obese):  4. MUHO  (Metabolically unhealthy obese): | In overall animal dietary pattern )ADP( was associated with MHO and  MUHO phenotypes. |
| 4 | ^(69)^Farhadnejad H, et al. (2019) | Cross-sectional | 3218  (aged ≥20 years/ Iranian people) | Dietary Approach to Stop Hypertension  (DASH) diet | Valid and reliable FFQ. | metabolically healthy obesity (MHO)  metabolically healthy obesity / )MUHO(. | Compared to MHO, greater adherence to the DASH diet was associated with 21% lower odds of MUH. There is positive association of DASH diet with healthy metabolic status. |
| 5 | ^(20)^Naja F, et al. (2020) | Cross-sectional | 305  (18 years old or older / Lebanese adults) | The unhealthy lifestyle pattern  The healthy lifestyle pattern | 80-item FFQ | metabolically healthy obesity (MHO) | The healthy lifestyle pattern and traditional-Lebanese pattern was associated with higher odds of MHO. |
| 6 | ^(58)^N. Slagter S, et al. (2018) | Cohort | 9270  (30–69 years old/western European) | Savory snacks and sweets pattern  Meat and alcohol pattern  Bread, potatoes and sweet snacks pattern  Fruit, vegetables and fish” pattern. | Self-administered FFQ. | metabolically healthy obesity (MHO)  metabolically healthy obesity / )MUHO(. | The healthier diet characterized by ‘fruit, vegetables and fish 'positively related to MHO. While, the carbohydrate-rich ‘bread, potatoes and sweet snacks’ dietary pattern was found to counteract MHO. |
| 7 | ^(64)^Arenaza L, et al.  (2019) | Cross-sectional | 137  (12–17 years old/ European adolescents) | Mediterranean dietary pattern (MDP) | Two non-consecutive 24 hours recalls were used for dietary intake assessment and the adherence to the MDP was calculated using (MDP score) (range 0–9). | metabolically healthy obesity (MHO)  metabolically healthy obesity / )MUHO(. | Adherence to the Mediterranean dietary pattern might be beneficial to maintain MHO. |
| 8 | ^(70)^Soltani S, et al. (2018) | Cross sectional | 403  (older than 18 years/ Iranian adults ) | Inflammatory potential of diets | 3-day, 24-h  Food recall. Empirical Dietary Inflammatory Pattern (EDIP). | Unhealthy phenotype. | A higher pro-inflammatory diet, evaluated based on food group intakes, was associated with higher odds of having an unhealthy metabolic phenotype in overweight/obese adults. |
| 9 | ^(67)^Y-M Park et al. (2016) | Cohort | 1739  (20–88 years old/ participants  of the National Health and Nutrition Examination Survey (NHANES)) | Mediterranean  Diet score (MDS). | FFQ and the 24-h dietary recall | Metabolically healthy  obese (MHO) | In overall, MHO individuals showed higher ratio of MUFA to SFA, and consumed less red meats and dairy products resulting in higher MDS for those components, compared with MUO individual's consumption of poultry and alcohol was higher with increasing tertile of MDS only in MUO individuals. Also, Higher adherence to Mediterranean diet was associated with a lower risk of all-cause mortality in the metabolically healthy obese (MHO) compared to metabolically unhealthy obese (MUO). |
| 10 | ^(62)^Matta J, et al. (2016) | Cross-sectional | 196  (MUHOv/O: 42.7 ± 15.7 years old and MHOv/O: 39.2 ± 13.0 years old / Lebanese adults) | Fast food/dessert pattern, Traditional-Lebanese pattern High-protein pattern | FFQ (61 items) | Metabolically Healthy Overweight and Obesity (MHOv/O)  Metabolically unHealthy Overweight and Obesity  MUHOv/O: | There's not significant associations were observed between the Fast Food/Dessert and the high-protein patterns with MHO. Participants with higher adherence to the Traditional-Lebanese dietary pattern had higher odds of belonging to the MHO phenotype. |
| 11 | ^(60)^K. Bell L, et al. (2015) | Cross-sectional | 2415  (45 years old and older/Australian adults) | Red meat and vegetable dietary pattern  Refined processed pattern  Healthy pattern | 24-hr dietary recalls were used to assess intakes. At least eight days following the first 24-hr recall, participants were invited for a second 24-hr recall over the phone | metabolically healthy obesity (MHO)  metabolically healthy obesity / )MUHO(. | A healthier dietary pattern plays a role in a metabolic and BMI phenotype. Indeed, for every one standard deviation increase in the Healthy dietary pattern, the odds of having a more metabolically healthy profile increased by 16% |
| 12 | ^(86)^V. Nerurkar p, et al. (2021) | Cross-sectional | 2201  (18 years or older) | Coffee consumption, plasma folate and vitamin B12 | FFQs and 24-hr dietary recalls based on NHANES.  Serum folate and vitamin B12 were analyzed using the Bio-Rad Laboratories “Quanta phase II Folate/ Vitamin B12” radio-assay kit. | metabolic syndrome  (MetS ) | Increased coffee consumption was significantly associated with MUHP among overweight individuals. Similarly, high plasma folate levels were associated with MUHP among the overweight and obese population. In contrast, lower plasma B12 levels were associated MUHP but not with obesity. |
| 13 | ^(126)^Moslehi N, et al. (2020) | Cohort | 1114  (Age ≥19 years/ Iranian adults) | Flavonoids and carotenoids. | 168-food items-FFQ | metabolic syndrome (MetS ) | Higher intakes of flavonoids and their individual classes may contribute to the lower risk of a metabolic unhealthy phenotype in both normal weight and overweight/obese adults. Flavonoids may have more favorable metabolic health effects than carotenoids. |
| 14 | ^(13)^Cordeiro A, et al. (2020) | Cross-sectional | 232  (21–59 years old/ Brazilian people) | Serum vitamin D. | HPLC with ultraviolet (UV) detector (Chromsystems, BioSysLtda, Rio de Janeiro, Brazil) | MHO (Metabolically healthy obese):  MUHO  (Metabolically unhealthy obese) | The prevalence of inadequacy of serum concentrations of 25(OH) D and greater severity of this deficiency in individuals with metabolically unhealthy obesity (MUHO) phenotype was high. Low serum concentrations of this vitamin were associated with metabolic disorders. |
| 15 | ^(73)^Qorbani M, et al. (2020) | Cross-sectional | 408  (7-18 years old/ Iranian students) | Daily consumption of certain food groups; and dietary behavior | Information about breakfast consumption  , eating speed  consumption at least 3 meals/day, consumption  of sugar-sweetened beverages, junk foods (including  Salty snack, Sweet consumption, and Fast food), fruits,  and vegetables was filled out by all students. | metabolic syndrome  (MetS ) | Daily consumption of salty snack was more frequent in the metabolically unhealthy obese (MUO) group. |
| 16 | ^(74)^ Mirmiran P, et al. (2020) | Cohort | 1114  (≥19 years old/ Iranian people) | Food items | 168-item FFQ | unhealthy phenotype | Higher intakes of magnesium, dairy products, poultry, apples/pears, citrus fruits, and tea/coffee decreased the risk of developing unhealthy phenotype. While, higher intakes of fast foods, organ meats, and potatoes increased the risk. |
| 17 | ^(35)^Ramos-Lopez O, et al.  (2019) | Cross-sectional | 298  (MHO: 42.4±10.3 years old and MUHO: 48.0±9.9 years old/ Spanish adults) | Dietary intake | 137-item  FFQ. | metabolically healthy phenotype (MHP )  metabolically unhealthy phenotype( MUP) | Total dietary fat was positively associated with MUHO. |
| 18 | ^(85)^Suarez‐Ortegon et al. 2019 | Cross-sectional | 312 (4-14 years old/ Spanish population) | Iron status | Serum ferritin was examined using the micro particle enzyme immunoassay (AxSYM;Abbot Laboratories, Abbot Park, Illinois). Serum transferrin and blood hemoglobin (EDTA sample; Beckman Coulter, Hialeah, FL) were measured by routine laboratory tests. | Cardio- metabolic healthy/ unhealthy | MUHO are associated with higher ferritin and hemoglobin levels in pre-pubertal children. |
| 19 | ^(71)^Nasreddine L, et al. (2018) | Cross-sectional | 1047  (10-19 years old/ Arabian adolescent) | Daily intake of Certain food groups | The Arabic version of the Global School Health Survey questionnaire was administered to collect data on lifestyle, dietary intake, and other health related behaviors. | MHO (Metabolically healthy obese):  MUHO  (Metabolically unhealthy obese) | Vegetable intake were found to be significantly associated with MHO in the study population, particularly in boys. |
| 20 | ^(72)^Roberge, et al. (2019) | Cohort | 564  (8-10 years old/ Canadian children) | Intake of macronutrient and food group | using the average of three 24-hour diet recalls. | MHO (Metabolically healthy obese):  MUHO  (Metabolically unhealthy obese) | Study showed that metabolically healthy obese children who ate fewer daily portions of fruits and vegetables were more likely to become MUHO two years later. |
| 21 | ^(79)^Aldhoon-Hainerova I, et al.  (2017) | Cross-sectional | 710  (13.0-17.9 years old/ Caucasian adolescents) | Macronutrients -carbohydrate, fat and protein (grams per day), fiber (grams per day) and calcium (milligrams per day). | Dietary intake was assessed using 3 dietary records (2 weekdays and 1 weekend day). | MHO (Metabolically healthy obese):  MUHO  (Metabolically unhealthy obese) | MHO boys had significantly lower protein intakes, while MHO girls had significantly higher intakes of total energy, carbohydrate, fat and calcium as compared to those with MUHO. |
| 22 | ^(27)^Li L, et al. (2016) | Cross-sectional | 1213  (6 –18 years old/ Beijing children) | Dietary records. | Dietary records including 10 items were assessed with the question, “What is your frequency of food intake?” Response options were ranged from seldom or never to every day | MHO (Metabolically healthy obese):  MUHO  (Metabolically unhealthy obese) | A healthy diet habit such as reduced consumption of soft drinks would have independently impact on metabolically healthy obesity (MHO) status. |
| 23 | ^(75)^W. Kimokoti R, et al. (2015) | Cross-sectional | 4855  (≥ 45 years old/ American men) | Daily intake | 107-item semi quantitative FFQ. | metabolic syndrome  (MetS ) | Healthy obesity in men is not associated with a healthier diet. |
| 24 | ^(57)^M. Camhi S, et al. (2015) | Cross-sectional. | 1235  (12–18 years and adults 19–85 years old/ American people) | Healthy eating index | In-person 24-hour recall collected using the USDA's Automated Multiple Pass Method. Healthy Eating Index 2005 (HEI-2005) scores were calculated using the Center for Nutrition Policy and Promotion and National Cancer Institute HEI SAS code. The food group standards are based on the recommendations found in MyPyramid. | MHO (Metabolically healthy obese): | MHO adolescents and women 19–44 years have better dietary compliance to the USA guidelines (HEI) when compared with MUHO. |
| 25 | ^(91)^M. Phillips C, et al. (2013) | Cross-sectional | 2047  (men and women 45–74 years old/ Caucasian  people) | Total dietary intake and Alcohol consumption. | A modified version of the self-completed EPIC FFQ. | metabolic health definitions based on a Several cardio metabolic abnormalities | Total and oily fish consumption and dietary fat composition were not different between MHO and MUO subjects. Also alcohol behavior revealed few differences. |
| 26 | ^(30)^ Park JM, et al.  (2012) | Cross-sectional | 3050  (> 20 years old/ Korean adults) | Intake of total energy | 24-h recall.  Regular diet was defined as having 3 meals a day for 2 days | Metabolically obese normal weight (MONW) | Reduced intake of carbohydrates and carbohydrate snacks were associated with a lower prevalence of metabolically MUNW in females. |
| 27 | ^(87)^A. Abdurahman A et al.  (2019) | Cross-sectional | 300  (19–59 years old/ Iranian people) | Dietary inflammatory index (DII) | Semi-quantitative FFQ (168 items) | MHO (Metabolically healthy obese)  MUHO  (Metabolically unhealthy obese) | Higher dietary inflammatory index (DII) scores were positively associated with the MUHO phenotype. A more pro-inflammatory diet is a potential risk factor for MUHO phenotype.  High consumption of the pro-inflammatory food components such as saturated fat, red meat and refined carbohydrates has been associated with several metabolic alterations and MetS. |
| 28 | ^(68)^Konieczna J, et al., (2020) | Cohort | 5801  (55-80 years old men and women aged 60-80 years old)/(Spanish people) | Mediterranean diet (MD) | 14-item Validated Mediterranean diet adherence screener (MEDAS)  And 137-item FFQ (dietary composition of macro and micronutrients) | metabolic syndrome  (MetS ) | Increase in adherence to - MDP has a potential to exert differential health benefits for all four obesity phenotypes, promoting metabolic health improvement in MUHO and MUNW older men and women at high CVD risk, as well as protecting against metabolic health deterioration in MHO and obesity incidence in, MHNW people. |
| 29 | ^(84)^Arsic A, et al. (2021) | Cross-sectional | 171 (30-65 years old/ European adults) | Polyunsaturated fatty acids (PUFA) intake | Two 24-h dietary recalls collected on one working  134 day and one weekend day | MHO (Metabolically healthy obese):  MUHO  (Metabolically unhealthy obese)  metabolically unhealthy nonobese (MUHNO) | Polyunsaturated fatty acids (PUFA) intake differ between metabolically healthy and unhealthy adults. Indeed, dietary intake of total PUFA and n-6 PUFA in MUHO persons was higher when compared with metabolically healthy obese and non-obese persons. |
| 30 | ^(63)^Barrea L, et al. (2021) | Cross-sectional | 94  (18–30 years old/ Italian PCOS Patients) | Mediterranean Diet  total energy intake | Prevention  with Mediterranean Diet (PREDIMED) questionnaire, a brief 14-item questionnaire, and  a face-to-face seven-day food records. | MHO (Metabolically healthy obese):  MUHO  (Metabolically unhealthy obese) | Findings of this study evidenced that MUO-PCOS patients presented also lower adherence to the MDP, compared to their MHO-counterpart. |
| 31 | ^(65)^Cobos-Palacios L, et al. (2022) | Cross-sectional | 144  (4–9 years old/ Spanish children) | Adherence to Mediterranean Diet (MedDiet) | 3-day dietary record (2 workdays and 1 weekend day), containing detailed information about food composition and cooking recipes over 72-h  and a FFQ were completed in every visit.  Adherence to MedDiet was evaluated using a validated questionnaire of 14 items related to food consumption frequency. | MHO (Metabolically healthy obese):  MUHO  (Metabolically unhealthy obese) | Metabolically healthy pre-pubertal population with obesity (MHOPp) subjects showed increased resistin serum levels after 12 and 24 months of MedDiet and PA. |
| 32 | ^(138)^ Tanisawa, K, et al. (2022) | Cross-sectional | 2,170  (1,354 men, 816 women (≥40 years old/ Japanese adults) | Dietary intake of 58 food and beverage items | Validated brief self-administered diet history questionnaire (BDHQ) | MHNO (Metabolically healthy nonobese)  MHO (Metabolically healthy obese)  MUHO  (Metabolically unhealthy obese) | Healthy dietary pattern positively associated with the prevalence of metabolically healthy phenotypes regardless of obesity status. Also reported that the alcohol dietary pattern was inversely associated with the prevalence of metabolically healthy phenotypes regardless of obesity status. |
| 33 | ^(66)^Cobos-Palacios L, et al. (2021) | Cross-sectional | 158  (65–87 years old/ Spanish population) | Adherence to Mediterranean Diet (MedDiet) | Validated 14-item FFQ.  non-consecutive, three-day dietary record (two workdays and one weekend day) | Metabolically healthy overweight/obese elderly (MHOe) | Ongoing MedDiet intake and regular physical activity (PA) can be considered preventative treatment for metabolic diseases in MHOe subjects. |
| 34 | ^(82)^H. Mazri F, et al. (2021) | Cross-sectional | 299  (40.3 ± 6.9 years old/Malaysian people) | Macro-nutrient and energy intake. | Validated 7-day Dietary History Questionnaire (DHQ) | metabolically healthy obese (MHO) | Consuming less energy earlier in the day and more energy and carbohydrate later in the day associated with a greater risk of MUHO. |
| 35 | ^(83)^L. Hankinson A, et al. (2013) | Cross-sectional | 775  (40–59 years old/ American adults) | Total energy intake, macro-/micronutrients, and  alcohol intake. | In-depth multi-pass 24-hour recall method | Metabolically healthy | Diet composition, measured by food group and macro-/micronutrient intake, was not associated with obesity phenotype. |
| 36 | ^(78)^M. Camhi S, et al. (2015) | Cross-sectional | 46  (19– 35 years old/ African, American and Caucasian women) | Dietary intake | The 2005 Block-110 item FFQ | MHO (Metabolically healthy obese) | MHO women also demonstrated healthier overall dietary intake with higher intake of vegetables, fiber, and lower intake of saturated fat and dairy products. |
| 37 | ^(61)^LM Pereira D, et al.  (2019) | Cross-sectional | 896  (20–59 years old/ Brazilian adults) | Unhealthy pattern  Traditional pattern  Healthy pattern | 95 food items FFQ | metabolically healthy / unhealthy | Unhealthy pattern was positively associated with the MHO and MUHO phenotypes. The more energy dense dietary pattern was related to excess weight phenotypes. moreover, the healthier dietary pattern was also related to these phenotypes, probably due to underhand over-reporting of foods by overweight individuals and reverse causality. |
| 38 | ^(56)^Fernández-Verdejo R, et al.  (2020) | Cross-sectional | 2287  (18 to <65 years old/ Chilean people) | Fruits/vegetables consumption | Questionnaires about the amount–in 80-g portions–and frequency of  consumption in a standard week | metabolically healthy / unhealthy | In normal weight subjects the highest quartile of fruits/vegetables consumption was associated with reduced odds of having an unhealthy phenotype in all models. Finally, fish/seafood consumption 1 to <3 times/month was associated with reduced odds of having an unhealthy phenotype. |
| 39 | ^(129)^Rouhani P, et al. (2023) | Cross-sectional | 203  (Iranian school adolescents (102 girls and 101 boys)) | Dietary intakes | food frequency questionnaire (FFQ) | metabolically healthy obese (MHO) and metabolically unhealthy obese (MUO) | There is a strong association between following a diet high in fat and sodium and an increased likelihood of being metabolically unhealthy overweight (MUO) in Iranian teenagers, particularly among boys and those who are obese. |
| 40 | ^(76)^Tirani SA, et al. (2022) | Cross-sectional | 203  (Iranian adolescents, 101 boys and 102 girls) | Dietary dairy intakes | food frequency questionnaire (FFQ) | metabolically healthy obese (MHO) and metabolically unhealthy obese (MUO) | Higher dairy intake was linked to lower odds of MUO among Iranian adolescents, particularly in girls and overweight individuals. |
| 41 | ^(132)^Rezazadegan M, et al. (2022) | Cross-sectional | 203  (Iranian overweight/  obese adolescents 102 girls and 101 boys (12– <18 years  old) | dietary acid load (DAL) | food frequency questionnaire (FFQ) | Metabolically healthy obesity (MHO) or metabolically unhealthy obesity (MUO) | Higher DAL levels may increase the likelihood of developing the MUO phenotype in Iranian overweight or obese adolescents. |
| 42 | ^(15)^Khadem A, et al. (2022) | Cross-sectional | 217  (women, aged 18–48 years old) | Lifelines Diet Score (LLDS) and Dietary intake | food frequency questionnaire (FFQ) | metabolically healthy/unhealthy overweight and obesity (MHO/MUHO) | Research suggests that persons who stick to the LLDS have a lower risk of becoming metabolically unhealthy (MUH). |
| 43 | ^(77)^Ooi DS, et al. (2022) | Cohort | 52  (Children and  adolescents with obesity, and aged 7 to 19 years old) | dietary food groups, nutrient intakes and eating behaviors | Three-Factor Eating Questionnaire- Revised 18-item version (TFEQ-R18) | Metabolically healthy obesity (MHO) or metabolically unhealthy obesity (MUO) | A healthier dietary composition and improved eating habits may contribute to better metabolic outcomes in obese children and adolescents. |
| 44 | ^(122)^Leone A, et al. (2022) | Cross-sectional | 2,115  (obese women, aged ≥18 years old) | Mediterranean Diet | MEDiterranean Diet Adherence Screener (MEDAS) | Metabolically Healthy Overweight (MHO), and Metabolically Unhealthy Overweight (MUO) | Postmenopausal women who followed the Mediterranean diet had improved metabolic health. |
| 45 | ^(137)^Tang D, et al. (2022) | Cross-sectional | 99 556  (Subjects from seven ethnic groups in Southwest China) | alternative Mediterranean (aMED) diet and Dietary Approaches to Stop Hypertension (DASH) diet | food frequency questionnaire (FFQ) | Metabolically healthy obesity (MHO) | This large population-based study found that the DASH diet, rather than the aMED diet, reduces obesity risk and provides metabolic benefits. |
| 46 | ^(123)^Mohammadi S, et al. (2022) | Cross-sectional | 203  (Iranian adolescents (102girls and 101 boys) aged12–18 years) | Mediterranean diet | food frequency questionnaire (FFQ) | metabolically healthy obese (MHO) and unhealthy obese  (MUO) | The study demonstrated a negative connection between the Mediterranean diet and the risk of MUO among Iranian adolescents. |
| 47 | ^(124)^Mirzaei S, et al. (2022) | Cross-sectional | 203  (Iranian adolescents (102 girls and 101 boys) ages 12 to <18 years old) | Mediterranean and Western dietary | food frequency questionnaire (FFQ) | metabolically healthy obese (MHO) and unhealthy obese (MUO) | The study demonstrated an inverse relationship between the Mediterranean-like dietary pattern and the odds of MUO in Iranian overweight/obese adolescents, while the Western dietary pattern was linked to an increased likelihood of being MUO. |
| 48 | ^(125)^Golzarand M, et al. (2023) | Cohort | 1303  (Iranian adults (aged ≥ 19  years old)) | Mediterranean Dietary  (MeDi) | food frequency questionnaire (FFQ) | metabolically healthy normal weight (MHNW) and metabolically healthy overweight/obesity (MHOW/MHO) | High compliance with the MeDi was linked to a lower MUNW risk.  There is a negative correlation between these three dietary patterns and the prevalence of metabolically unhealthy conditions. |
