# Supplementary material for: Association of Genetic, Environmental, and Nutritional Factors With Metabolic Phenotypes of Obesity: A Scoping Review
Source: J Obes. 2025 Jul 2;2025:8472196. doi: 10.1155/jobe/8472196 (PMC12259317; doi:10.1155/jobe/8472196)
Supplement: Supporting Information — Supporting Table-4: Characteristics of the included studies about environmental factors. [file 8472196.f4.docx]

Table S4- characteristics of included studies about environmental factors

| **ID** | **Author**  **(year)** | **Study design** | **Sample size**  **(age/ race)** | **Environmental factor** | **Method of assessment of Environmental factor** | **definition Obesity phenotype and**  **metabolic criteria** | **Main finding** |
| --- | --- | --- | --- | --- | --- | --- | --- |
| 1 | ^(83)^L.Hankinson A, et al (2013) | Cross-sectional | 2195  (40–59 years old/ American people) | Lifestyle behaviors (sleep duration, television viewing time, leisure-time, work-related physical activity, and smoking) | Interviewer-administered questionnaire | MHO (Metabolically healthy obese) | Sleep duration was associated with obesity phenotype in women. While, other activity behaviors factors, including television viewing time, other sedentary activity, and physical activity levels were not significantly related to obesity phenotype. There were no significant differences in sleep duration, television viewing time, other sedentary activity, and physical activity levels between obesity phenotypes in men. |
| 2 | ^(78)^M. Camhi S, et al.  (2015) | Cross-sectional | 46  (19– 35 years old/  African American and Caucasian women) | Lifestyle behaviors (Physical activity and Sedentary behavior) | Physical activity Questionnaires | MHO (Metabolically healthy obese) | MHO women spent less time per day in sedentary behavior and more time per day in light physical activity, resulting in an overall higher daily energy expenditure compared with MUHO. |
| 3 | ^(45)^ Kanagasabai T, et al. 2017 | Cross-Sectional | 1777  (≥20 years old/Non-Hispanic White, Non-Hispanic Black, Mexican American, and Other) | Lifestyle behaviors and socio-demographic factors (Sleep Habits/ smoking status/ ethnicity/ education) | The Sleep Disorders questionnaires | Metabolically Healthy Obesity (MHO)  metabolically abnormal obesity (MAO). | MHO shows slightly better overall sleep quality, but neither sleep duration nor overall sleep quality was associated with the odds of MHO in fully adjusted models. However, regularly waking up during the night, feeling unrested during the day, feeling overly sleepy during the day, and having trouble falling asleep were associated with lower odds of MHO. MHO and MUHO individuals did not differ in education. MHO individuals tended to be more active, younger, female, and never smokers and were less likely to belong to the Non-Hispanic White ethnic group. |
| 4 | ^(90)^ Ryu JY, et al.  (2014) | Survey | 9077  (>= 20 years old / Korean adults) | Lifestyle behaviors (sleep duration) | Self-reported questionnaire | metabolically healthy non-obese (MHNO)  metabolically healthy obese (MHO)  metabolically unhealthy non-obese (MUNO)  metabolically unhealthy obese (MUO) | The results show sleep duration is significantly different according to body size phenotype in a Korean population, even after adjusting for various kinds of lifestyle and socio-economic factors. Metabolically unhealthy subjects also showed shorter sleep duration compared to metabolically healthy subjects. |
| 5 | ^(52)^ S. Dusanov, et al.  (2018) | Cross-sectional | 431  (161 men and 270 women/ Norway people) | Organic pollutants | Laboratory test. | metabolically healthy / unhealthy | In morbidly obese persons the odds of metabolic syndrome were increased with higher concentrations of dioxin-like and non-dioxin-like PCBs. |
| 6 | ^.(46)^ Lim HH, et al  (2018) | Cross-sectional | 3650  (1946 boys and 1704 girls: 12-18 years old/ Korean children and adolescents ) | Lifestyle behaviors and socio-demographic factors (sleep duration/ household income.) | Self-reported  questionnaire | metabolically healthy” metabolically unhealthy” | After controlling for confounding factors, sleep duration was independently associated with metabolic body size phenotype in children and adolescents. Also, MHNW had significantly higher household income than MUNW subjects. |
| 7 | ^(89)^ Ren H, et al. (2019) | Cross-sectional | 4149  (18-94 years old/ Chinese adults) | Lifestyle behaviors  (Sleep duration) | Self-reported  questionnaire | metabolically healthy obese (MHO)  metabolically healthy overweight/obesity (MHOO) | The prevalence of short sleep duration was higher (12.2% vs 9%) and long sleep duration was lower (66.6% vs 69.6%) in the MUHO group compared with the MHO group. |
| 8 | ^(56)^ Fernández-Verdejo R, et al.  (2020) | Cross-sectional | 2287  (18 to <65 years old/ Chilean people) | Lifestyle habits | Lifestyle habits questionnaires and  the global  physical activity questionnaire | metabolically healthy / unhealthy | Considering the overall population, the highest quartile of moderate-vigorous physical activity was associated with reduced odds of having an unhealthy phenotype. Also, former smoking was associated with reduced odds. In subjects with normal weight, the lowest tertile of alcohol intake was associated with increased odds of having an unhealthy phenotype. In subjects with overweight, former smoking was associated with reduced odds of having an unhealthy phenotype. Also, the lowest tertile of alcohol intake was associated with reduced odds of having an unhealthy phenotype. In the obesity category, the highest quartile of moderate-vigorous physical activity was associated with reduced odds of having an unhealthy phenotype, while the second quartile was associated with reduced odds. |
| 9 | ^(26)^ Li G, et al. (2020) | Cohort | 1475  (6–18 years old/ Chinese children) | Lifestyle behaviors and socio-demographic factors  (Physical activity, sleep duration,  mode of transportation to school,  annual household income, and parental education) | Lifestyle factors questionnaires | Metabolically healthy normal weight (MHNW) | This study exhibiting that lifestyle and socioeconomic factors were independent risk factors for the MUNW phenotype. Indeed, not only behavioral factors such as physical activity and fruit consumption, moreover socioeconomic components including parental education and household income have an independent impact on childhood MUNW status. Eventually, some early environments (i.e. birth weight) including their interaction with one another, play important roles in predicting the MUNW phenotype among children. |
| 10 | ^(47)^ Suebsamran P, et al.  (2021) | Cross-sectional | 19,640  (15 years and older/ Thai people) | Lifestyle behaviors and socio-demographic factors  (Physical activity/ Smoking/socio-economic/ living area/ education) | Global Physical Activity Questionnaire (GPAQ) | metabolically healthy (MH) and metabolically unhealthy (MUH). | Characteristics associated with MUH were being female, being older, having a low level of education, living in a rural area and currently smoking tobacco. In this population, Alcohol drinking and physical activity were not found to be associated with MUH. |
| 11 | .^(91)^ Phillips C, et al  (2013) | Cross-sectional | 2,047  (50-69 years old/ Irish people) | Lifestyle behaviors  (Physical activity/ smoking/ alcohol consumption) | International Physical Activity Questionnaire (IPAQ) | metabolically healthy obese (MHO)  (Metabolically unhealthy obese (MUHO)  Metabolically healthy normal weight (MHNW)  Metabolically unhealthy normal weight( MUNW) | Moderate and high levels of physical activity increase the likelihood of metabolically healthy obesity (MHO). Physical activity level, smoking status and alcohol consumption were not different between the MHNW and MUNO subjects. In the secondary analyses examination of physical activity measures, smoking behavior and alcohol consumption did not reveal any significant differences between the metabolically healthy and unhealthy participants, regardless of BMI. |
| 12 | ^(50)^ Gasull M et. al  (2017) | Cross-sectional | 860  (18-74 years old/ Spanish people) | Persistent Organic Pollutants (POP) | Serum POP concentration assays laboratory test. | metabolic syndrome  (MetS ) | Results supported the hypothesis that POP concentrations are associated with unhealthy metabolic phenotypes, not only in obese and overweight individuals but also (and probably more strongly) in normal-weight individuals. |
| 13 | ^(51)^ K.H. Ha et al  (2018) | Case-control | 50 case and 50 control  (Korean population) | Persistent organic pollutants (POP) | Serum concentrations of PCBs and OCPs were determined  Using high-resolution gas chromatography (HRGC) with high-resolution  mass spectrometry (HRMS; AutoSpec Premier, Waters Corp.,  Milford, MA, USA). | Metabolically healthy normal weight (MHNW)  Metabolically unhealthy normal weight( MUNW) | Increased serum POP concentrations may play an important role in the development of unhealthy metabolic phenotypes in lean people. |
| 14 | .^(54)^ Xu Y et al  (2020) | Cross-sectional | 1392  (mean age 43.1 ± 9.8 years, Chinese adults) | The urinary levels of 23 metals | The urinary levels of 23 metals were  determined by the inductively coupled mass spectrometer (ICPMS;  Agilent Technologies, 7700X, USA) | unhealthy metabolic phenotype | The results suggest that urinary zinc and zinc-copper ratio are positively associated with increased risk of unhealthy metabolic. |
| 15 | ^(49)^ Gauthier M et al  (2014) | Cross-sectional | 76  (mean of age : 57.6 years old (MHO) and 58.3 years old (MAO, Canadian population) | Plasma concentrations of 21 Persistent organic pollutants (POPs) | POPs were measured in a plasma sample (0.5 mL) collected after an overnight fast. | metabolically healthy obese (MHO)  / metabolically unhealthy obese (MUHO) | Study demonstrates that the metabolically healthy and abnormal phenotypes have distinct plasma POP profiles. Indeed, MHO individuals had significantly lower circulating levels of various classes of POPs than MUHO patients. |
| 16 | ^(27)^ Li L et al.  (2016) | Cross-sectional | 1213  (6-18 years old, Chinese children) | Lifestyle and socio-demographic factors  (Physical activities/ The socioeconomic factors included the parents education and vocation/ birth weight) | Questionnaire | metabolically healthy obesity based on cardiometabolic risk (MHO-CR)  metabolically unhealthy obesity based on cardiometabolic risk (MUO-CR) | Both genetic predisposition and environment factors and their interaction contribute to the prediction of MHO status. Current lifestyle and socioeconomic factors such as walking to school and consumption of soft, mother's education as well as early intrauterine nutrition environment (birth weight) could independently predict MHO. |
| 17 | ^(53)^ [Wang](https://pubmed.ncbi.nlm.nih.gov/?term=Wang+W&cauthor_id=34653859) w et al  (2022) | Case-control | 88  (young adults aged 18-26 years) | Short-term ozone exposure | - | - | Compared to normal-weight people, obese people, both with a metabolically healthy status, might be more susceptible to the negative effects of O3 on metabolic status, possibly through inflammatory indicators such as leptin, eosinophils, and MCP-1. |
| 18 | ^(92)^ Imbiriba L, et al.  (2020) | Cross-sectional | 2371  (49.6 ± 7.1 years old/ Brazilian people) | Job stress | Swedish Demand-Control-Support Questionnaire (DCSQ) | metabolically unhealthy obese (MUHO) | A high metabolic profile and low skill discretion were significantly correlated. The metabolic profile of individuals with obesity and the job stress domain was not significantly correlated in full models. |
| 19 | ^(48)^Bouhours-nouet N et al.  (2008) | Cross-sectional | 117 (French children aged 6-15 years old) | Birth weight | French health care system and were retrospectively obtained from the individual health book | - | High birth weight (HBW) contribute to so-called metabolically healthy obesity. |
| 20 | ^(142)^ Pizarro A et al.  (2013) | Cross-sectional | 229  (age of 11.65 (±0.73) years old, Portuguese youths) | Transport to and from school  (Walking to school) | Questionnaire | metabolic syndrome  (MetS ) | Exertions to increase and maintain walking to school may be particularly relevant, as it is likely to have a positive impact on children’s health and eventually decrease metabolic disorders. |
| 21 | ^(80)^ Llorente-Cantarero FJ et al. (2022) | Cohort | 275  (children (144 boys) with 9 ± 2 years old) | Physical Activity | Accelerometry assessment | (MHO) metabolically healthy obesity, (MUO) metabolically unhealthy obesity | Prepubertal MHO children show lower sedentary behavior, more activity, and improved metabolic profiles compared to MUO children. All children, especially girls, need to participate in more physical activity, both in terms of time and intensity, as it can improve metabolic health. |
| 22 | ^(81)^ Cai S et al. (2022) | Cross-sectional | 15,114  (Chinese children and adolescents aged 7-18 years) | Lifestyle factors  (sleep duration, screen time, physical activity) | Questionnaire | (MHO) metabolically healthy obesity, (MUO) metabolically unhealthy obesity, (MHOO) metabolically healthy overweight and obesity, and (MUOO) metabolically unhealthy overweight and obesity | MHO and MUO are spread among Chinese children and adolescents. Significant sex differences in obesity phenotypic prevalence and environmental factors indicate a gender-based approach to obesity management. |
